# Down‐regulation of miR‐146a‐5p and its potential targets in hepatocellular carcinoma validated by a TCGA‐ and GEO‐based study

**DOI:** 10.1002/2211-5463.12198

**Published:** 2017-02-20

**Authors:** Xin Zhang, Zhi‐hua Ye, Hai‐wei Liang, Fang‐hui Ren, Ping Li, Yi‐wu Dang, Gang Chen

**Affiliations:** ^1^ Department of PathologyFirst Affiliated Hospital of Guangxi Medical UniversityNanningChina

**Keywords:** expression, gene signature, HCC, hepatocellular carcinoma, microRNA, miR‐146a‐5p

## Abstract

Our previous research has demonstrated that miR‐146a‐5p is down‐regulated in hepatocellular carcinoma (HCC) and might play a tumor‐suppressive role. In this study, we sought to validate the decreased expression with a larger cohort and to explore potential molecular mechanisms. GEO and TCGA databases were used to gather miR‐146a‐5p expression data in HCC, which included 762 HCC and 454 noncancerous liver tissues. A meta‐analysis of the GEO‐based microarrays, TCGA‐based RNA‐seq data, and additional qRT‐PCR data validated the down‐regulation of miR‐146a‐5p in HCC and no publication bias was observed. Integrated genes were generated by overlapping miR‐146a‐5p‐related genes from predicted and formerly reported HCC‐related genes using natural language processing. The overlaps were comprehensively analyzed to discover the potential gene signatures, regulatory pathways, and networks of miR‐146a‐5p in HCC. A total of 251 miR‐146a‐5p potential target genes were predicted by bioinformatics platforms and 104 genes were considered as both HCC‐ and miR‐146a‐5p‐related overlaps. RAC1 was the most connected hub gene for miR‐146a‐5p and four pathways with high enrichment (VEGF signaling pathway, adherens junction, toll‐like receptor signaling pathway, and neurotrophin signaling pathway) were denoted for the overlapped genes. The down‐regulation of miR‐146a‐5p in HCC has been validated with the most complete data possible. The potential gene signatures, regulatory pathways, and networks identified for miR‐146a‐5p in HCC could prove useful for molecular‐targeted diagnostics and therapeutics.

**Abbreviations**
AUC: area under the curve
EMT: epithelial mesenchymal transition
FCs: fold changes
GEO: gene expression omnibus
HCC: hepatocellular carcinoma
KEGG: Kyoto Encyclopedia of Genes and Genomes
miRs: microRNAs
NLP: natural language processing
ROC: receiver operator characteristic
TCGA: The Cancer Genome Atlas

Hepatocellular carcinoma (HCC) is considered to be the fifth most frequent cancer globally and takes the third place for cancer‐related mortality 1, 2. However, many patients are diagnosed at advanced stages, and recurrence and metastasis remain the main challenge for HCC treatment 3. Therefore, it is of utmost urgency to find novel diagnostic and prognostic biomarkers for HCC.

MicroRNAs (miRs) are an ample variety of short, noncoding RNA molecules of 18–25 nucleotides, which mediate numerous cellular processes, such as cell proliferation, migration, and apoptosis 4, 5. Among them is miR‐146a‐5p, which locates on human chromosome 5q34 and is thought to be actively involved in multiple oncological processes of HCC, such as antitumor immune suppression 6, metastasis 7, and angiogenesis 8. Our previous work 9 has demonstrated that the down‐regulated miR‐146a‐5p expression is associated with the carcinogenesis and deterioration of HCC and that miR‐146a‐5p might be a tumor‐suppressive microRNA of HCC. Nevertheless, the precise molecular mechanisms of miR‐146a‐5p in HCC remain largely unknown and obscure.

Believed to be promising in cancer diagnostics and prognosis predicting, gene signatures help to provide the molecular backgrounds, regulatory pathways, and networks of cellular activities in HCC 10. Cases in point are resources and techniques as follows: Gene Expression Omnibus (GEO) Database stores public array‐ and sequence‐based functional genomics data, which allows users’ query and downloading of experiments and gene expression profiles 11. Meanwhile, The Cancer Genome Atlas (TCGA) is one prominent example of the renowned public databases which contains the genetic information of various cancers. Furthermore, natural language processing (NLP) is a booming technique which teaches computers to comprehend and to sort out natural language by algorithms and programs, enabling researchers to retrieve papers on certain topics of interest and to analyze data automatically 12.

A succession of resources and techniques in bioinformatics and computational biology were applied in the study, which includes GEO and TCGA data aggregation, comprehensive meta‐analyses, NLP analysis, target genes prediction, analytic integration, and bioinformatics analyses. We aimed to validate the down‐regulation of miR‐146a‐5p in HCC with the most complete data currently available and to present the audience with the intriguing gene signatures, regulatory pathways, and networks of miR‐146a‐5p in the carcinogenesis, metastasis, prognosis, recurrence, survival, and drug‐resistance (sorafenib and bevacizumab) of HCC.

## Materials and methods

**Figure 1:**
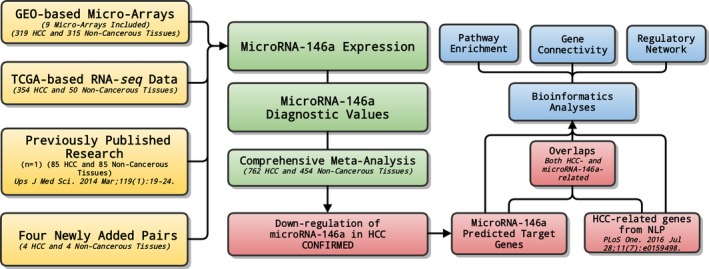
General flow chart. The present study is composed of several procedures sequentially; that is, GEO‐based verification of clinical values, TCGA‐based data aggregation of RNA‐seq, comprehensive meta‐analyses, and multiple bioinformatics analyses.

The present study consists of several processes sequentially (Fig. 1), that is, GEO‐based clinical values verification, TCGA‐based RNA‐seq data aggregation, comprehensive meta‐analyses based on GEO, TCGA and literature data, and multiple bioinformatics analyses.

### Clinical value verification of miR‐146a‐5p expression in HCC based on GEO datasets

All the functional genomics data of miR‐146a‐5p were requested and assembled from the GEO Database (http://www.ncbi.nlm.nih.gov/geo/) with the closing date of 10 September 2016. The search strategy formulated in the GEO datasets (http://www.ncbi.nlm.nih.gov/gds/) was as follows: (malignan* OR cancer OR tumor OR tumour OR neoplas* OR carcinoma) AND (hepatocellular OR liver OR hepatic OR HCC). Inclusion criteria were listed below: (a) HCC tissues were included in each dataset with each group containing more than two samples, regardless of the inclusion of adjacent noncancerous tissues (or healthy liver tissues); (b) the dataset sample organism was Homo sapiens; (c) the expression data of miR‐146a (hsa‐miR‐146a or hsa‐miR‐146a‐5p) from the experimental and control groups could be provided or calculated. Meanwhile, the following conditions might cause the exclusion of related datasets: (a) datasets without information on miR‐146a‐5p; (b) datasets without complete data for analysis; (c) samples based on cell lines; (d) not all the subjects of the included studies were human; or (e) miR‐146a‐5p was determined in the HCC patients without a comparison. Expression values of miR‐146a‐5p and sample size in both test and control groups were calculated. Moreover, means and standard deviations of these values were extracted to estimate the different levels of miR‐146a‐5p in case and control groups by using Review Manager 5.3 with random‐effects model. The chi‐square test and the *I* ^2^ statistics were applied to evaluate the heterogeneity across studies. It was considered to be heterogeneous when the *P* value <0.05 or *I* ^2^ > 50%. Furthermore, SMD and its 95% CI were pooled to assess the stability of the analysis. It was considered to be statistically significant if the corresponding 95% CI for the pooled SMD did not overlap 1 or ‐1. Additionally, sensitivity analysis was conducted by eliminating each study to evaluate the source of heterogeneity.

### RNA‐seq data aggregation based on TCGA database

From the TCGA (http://cancergenome.nih.gov/), we downloaded and extracted the data of miR‐146a‐5p expression from miRNASeqV2 (level 3), on 15 July 2016, through bulk download mode. MiR‐146a‐5p expression data were presented as upper quartile normalized Expectation‐Maximization (RSEM) count estimates 13, 14 by using the ‘rsem.gene.normalized_results’ file type. Related data were processed without further transformation, except that some values were rounded off to integers. The expression data between HCC and adjacent normal liver tissues were compared by limma package in R. Fold changes (FCs) were calculated as HCC vs. normal liver tissue. It would be considered as statistically significant if a FC value was <0.5 or >2 and with the *P* value <0.05 in Student's *t*‐test.

### Comprehensive meta‐analysis based on GEO, TCGA, and literature data

Comprehensive meta‐analyses were performed based on the data gathered from GEO, TCGA, and relevant literature. Related studies were selected by comprehensively searching through the online databases PubMed, Embase, Web of Science, Wiley Online Library, Cochrane Library, Science Direct, Chinese WanFang Database, Chinese VIP Database, Chinese Biomedical Literature Database, and Chinese CNKI Database up to 15 July 2016, independently. The following combination of keywords and entry words was employed: (a) (miR‐146a OR miRNA‐146a OR microRNA‐146a OR miR146a OR miRNA146a OR microRNA146a OR ‘miR 146a’ OR ‘miRNA 146a’ OR ‘microRNA 146a'OR miR‐146a‐5p OR miRNA‐146a‐5p OR microRNA‐146a‐5p); (b) (hepatocellular OR liver OR hepatic OR HCC); (c) (‘cancer’ OR ‘tumor’ OR ‘tumour’ OR ‘neoplas*’ OR ‘carcinoma’ OR ‘sarcoma’ OR ‘malignan*’). In addition, some references of relevant articles were manually searched for further studies. Whichever articles fulfilled all the following criteria were considered to be included: (a) There was no language restriction of the publications. (b) Patients with HCC were included. (c) The difference of miR‐146a‐5p expression between HCC and noncancerous controls was estimated. (d) If the study of the same patient cohort was published twice or more, only the most complete and recently published one would be included. Listed below were situations which caused the exclusion of related articles: (a) Reviews, letters, comments, case reports, editorials, expert opinions, and conference abstracts without original data were excluded. (b) Articles of experimental *in vitro* or *in vivo* studies were excluded. (c) We also excluded the studies with no information on the difference of miR‐146a‐5p between HCC and controls. (d) Since we had downloaded and evaluated TCGA data by ourselves, those studies based on TCGA data were excluded for the meta‐analysis based on literature.

The statistics were analyzed using spss 22.0 software (Armonk, NY, USA). The final data after calculation were presented as the means ± SD. Student's *t*‐test was used for a comparative analysis of two independent groups. To differentiate the expression data between controls and HCC tissues, the diagnostic value was identified using a receiver operator characteristic (ROC) curve. Any *P* value <0.05 denoted statistical significance. The meta‐analysis was performed using revman 5.3 (London, UK) 5.3. A standard mean difference (SMD) and a 95% confidence interval (CI) were utilized to measure continuous outcomes. Fixed or random‐effects models were applied to pool the effect sizes. Cochrane's *Q* test (Chi‐square test; Chi^2^) and inconsistency (*I*²) test were conducted to assess heterogeneity. A *P* < 0.05 or *I*² > 50% indicated significant heterogeneity, and a random‐effects model was applied. Otherwise, the fixed effects model would be selected. A funnel plot was generated to evaluate publication bias. A *P* value <0.05 was considered to indicate statistical significance.

### Bioinformatics analyses of miR‐146a‐5p and HCC

Generally, the bioinformatics analyses were conducted as formerly described 15, which included NLP procedure of HCC, prediction of miR‐146a‐5p target genes, and comprehensive analyses of the integrated genes.

#### NLP procedure of HCC

First of all, we conducted the document mining in PubMed, which included all related articles published between 1 January 1980 and 25 May 2015. The combination of keywords used was as listed: (hepatocellular carcinoma) AND (resistance OR prognosis OR metastasis OR recurrence OR survival OR carcinogenesis OR sorafenib OR bevacizumab) and (‘1980/01/01’ [PDAT]: ‘2015/05/25’ [PDAT]). A detailed list of relevant proteins and genes was created afterwards. Later on, obtained data went through multiple processes; that is, gene mention tagging and conjunction resolution by ABNER (http://pages.cs.wisc.edu/~bsettles/abner/) as well as gene name normalization according to Entrez Database developed by NCBI 16, 17. Finally came the statistical analysis featured by the hypergeometric distribution formulae shown, that is, $p=1-\sum_{i=0}^{k-1}p\left(i|n,m,N\right)$ and p(i|n,m,N)=(n!(N−n)!m!(N−m)!)/((n−i)!i!(n−m)!(N−n−m+i)!N!). *N* was defined as the total number of articles in PubMed. The letters *m* and *n* stood for the occurrence frequencies of relevant genes and HCC in PubMed, respectively. *K* was denoted as the co‐occurrence frequency of a certain gene and HCC at the same time in actual cases. Thus, we could calculate the probability of cocitation occurrence frequency greater than *k* under completely randomized conditions. The frequency of occurrence was output for each gene respectively: the higher frequency a certain gene demonstrated, the greater opportunity the gene harbored to be HCC‐related. The above methods for NLP procedure and corresponding results for HCC have been reported in our previous research article 15.

#### Prediction of miR‐146a‐5p target genes

A combination of 11 gene prediction platforms were used to predict the potential miR‐146a‐5p target genes; that is, TargetScan/TargetScanS 18, MirTarget2 19, DIANA‐microT 20, PicTar 21, PITA 22, MicroInspector 23, miRanda 24, RNA22 25, miTarget 26, RNAhybrid 27, and NBmiRTar 28. A predicted target gene would only be considered when nominated by at least four gene prediction platforms.

#### Comprehensive integration

We comprehensively analyzed HCC‐related genes from NLP procedure and potential miR‐146a‐5p target genes from prediction platforms and later generated the integration of the corresponding overlaps.

#### Enrichment pathway

We both mapped relevant genes into the Kyoto Encyclopedia of Genes and Genomes (KEGG) Pathway Database and calculated the enrichment *P* values of each pathway using genmapp v2.1 29.

#### Gene connectivity

The gene connectivity was calculated as a quantitative index to demonstrate the degree of interactions among genes and proteins.

#### Regulatory network

There were three different types of interaction relationships to construct the regulatory networks: (a) Available data from KEGG Database: we achieved the relevant data from KEGG Pathway Database and ported them into R (https://www.r-project.org/) with the keggsoap package (http://www.bioconductor.org/packages/2.4/bioc/html/KEGGSOAP.html) undergoing a genome‐wide interaction analysis (enzyme–enzyme relation, protein–protein interaction, and gene expression interaction). (b) Data from high‐throughput experiments: the MIPS Mammalian Protein–Protein Interaction Database (http://mips.helmholtz-muenchen.de/proj/ppi/) were employed for the protein–protein interactions data. (c) Existing data regarding gene interactions: data were processed with the hypergeometric distribution algorithm.

All the above factors and data were analyzed comprehensively and visualized by the medusa software (Cambridge, UK) in form of networks.

## Results

### GEO dataset verification of down‐regulated miR‐146a‐5p expression in HCC

**Figure 2:**
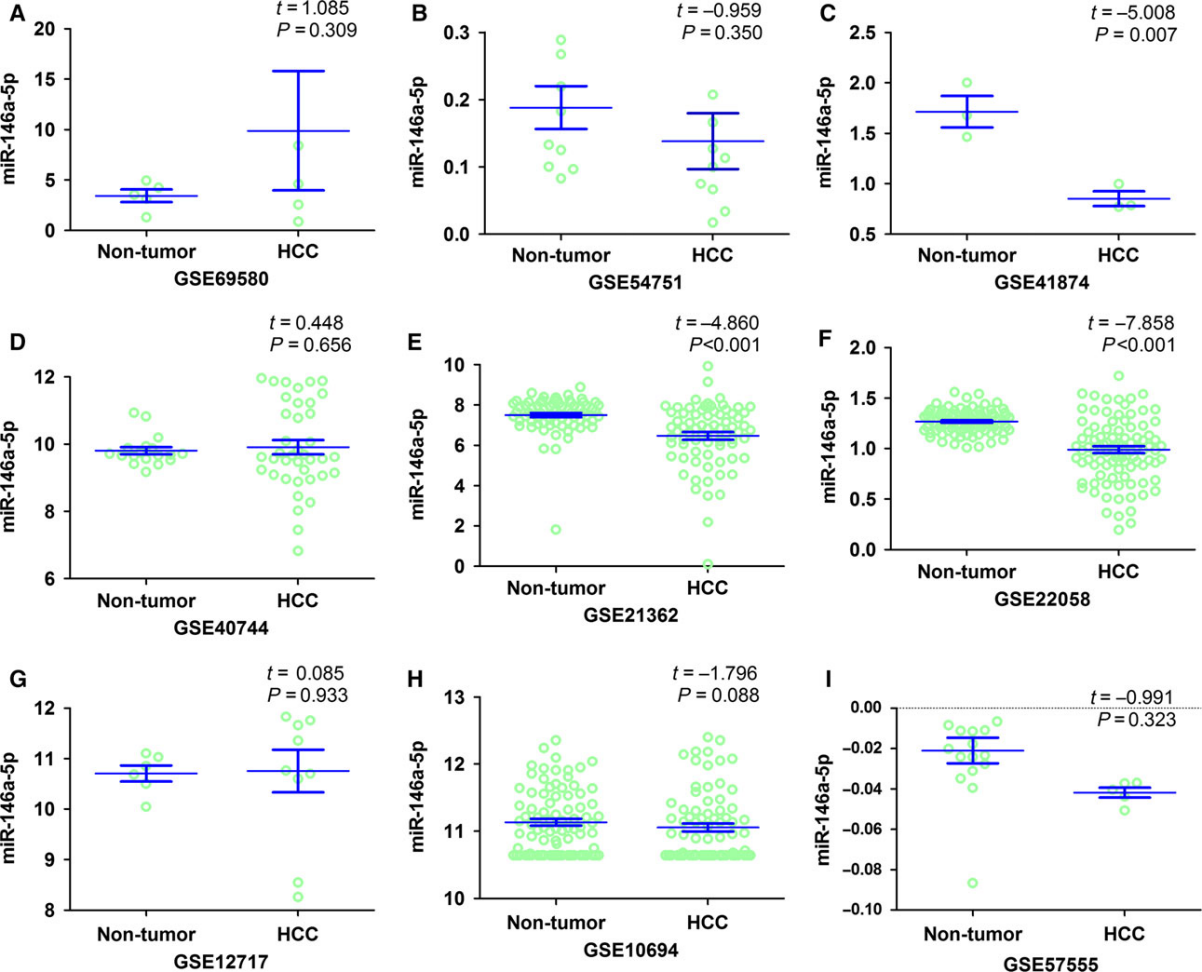
The expression data of miR‐146a‐5p in HCC in multiple microarrays from GEO. Nine microarrays were included in the analysis, among which three (GSE41874, GSE21362, GSE22058) proved it to be statistically significant that the miR‐146a‐5p expression was decreased in HCC tissues as compared to noncancerous tissues.

**Figure 3:**
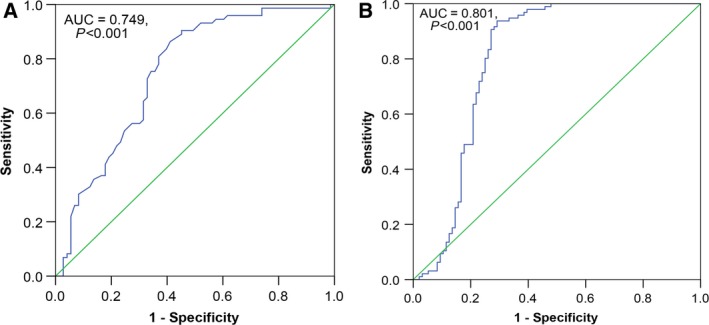
The ROC curve of miR‐146a‐5p for HCC in two microarrays. Two microarrays (GSE21362 and GSE22058) demonstrated the significant diagnostic value of miR‐146a‐5p in HCC. (A) GSE21362; AUC=0.749, 95% CI: 0.669–0.830, *P* < 0.001. (B) GSE22058; AUC=0.801, 95% CI: 0.731–0.872, *P* < 0.001.

**Figure 4:**
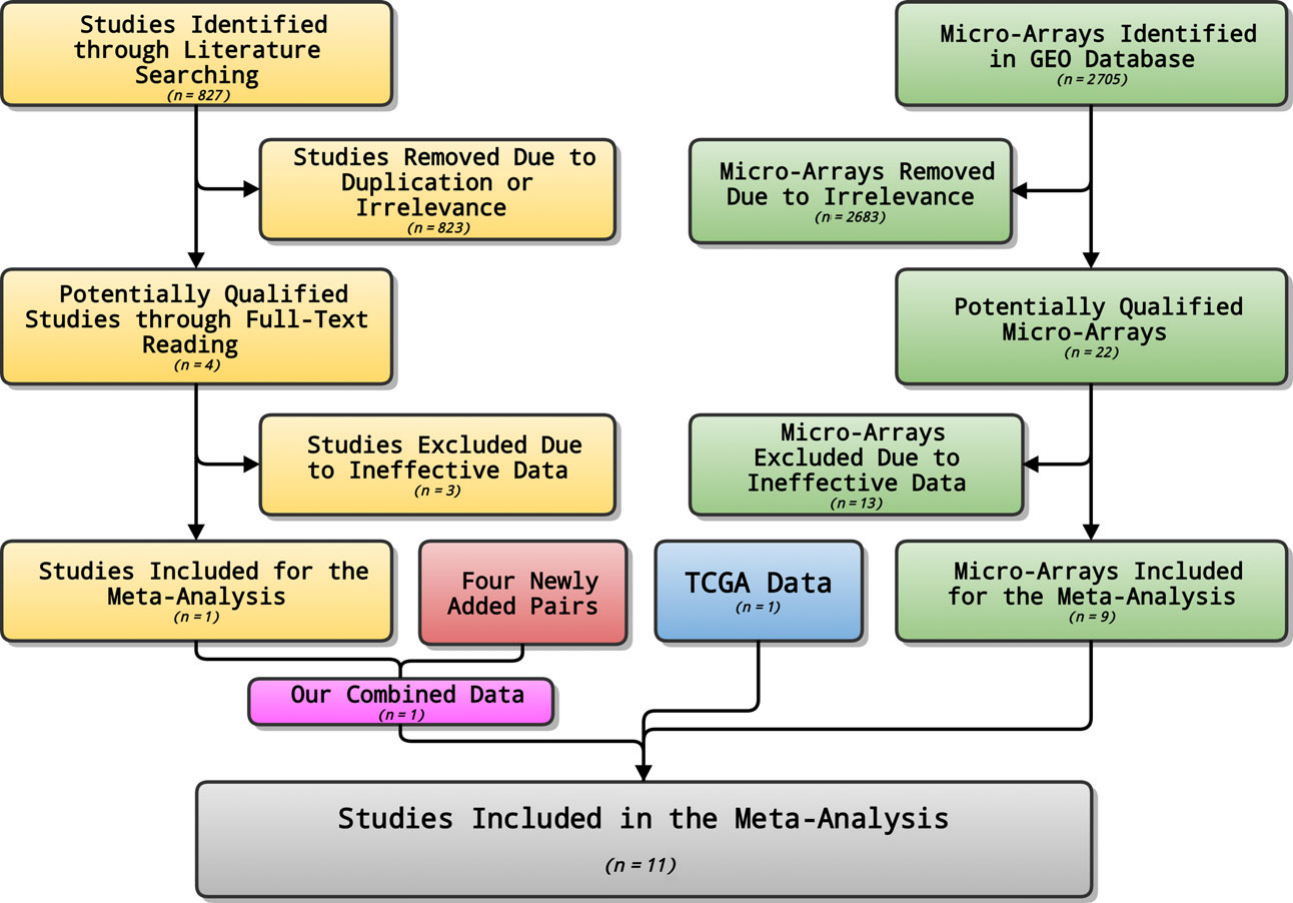
The flow chart of the meta‐analysis. We included a union of 762 HCC and 454 nontumor liver tissues for the meta‐analysis, which is from GEO database, TCGA dataset, our previous research article, and newly added samples and stands for the most complete data available.

**Figure 5:**
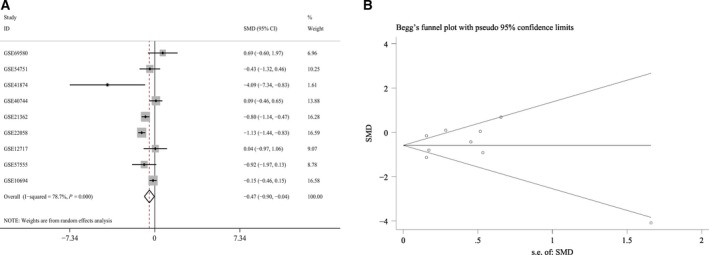
The forest plot and Begg's funnel plot of miR‐146a‐5p expression data in microarrays from GEO database. (A) The miR‐146a‐5p expression data of 319 HCC and 315 noncancerous liver tissues from GEO database were included. The pooled SMD of miR‐146a‐5p was −0.470 (95% CI:−0.902 to −0.038), *P* = 0.033) by the random‐effects model and the *P* value of the heterogeneity test was <0.001 (*I* ^2^ = 79%). (B) No publication bias was observed in the funnel plot (Begg's test: *P* = 0.917; Egger's test: *P* = 0.760).

A total of 2705 microarrays were identified during the primary searching, among which 22 were later downloaded from the GEO database (http://www.ncbi.nlm.nih.gov/geo/) after relevant assessments and evaluation. Eventually, nine microarrays were included in this part (GSE69580, GSE54751, GSE41874, GSE40744, GSE21362, GSE22058, GSE12717, GSE57555, and GSE10694, Fig. 2) after screening and inspection in accordance with the aforementioned inclusion criteria. Three microarrays (GSE41874, GSE21362, and GSE22058) demonstrated that the miR‐146a‐5p expression was significantly lower in HCC tissues than that in noncancerous tissues. Two microarrays (GSE21362 and GSE22058) (Fig. 3) displayed the significant diagnostic value of miR‐146a‐5p in HCC (AUC = 0.749, 95% CI: 0.669–0.830, *P* < 0.001; AUC = 0.801, 95% CI: 0.731–0.872, *P* < 0.001, respectively). The detailed information of the included studies for the meta‐analysis was summarized in Table 1 and the flowchart of this meta‐analysis was shown in Fig. 4. In short, 319 HCC and 315 nontumor liver tissues in GEO database were included for the later meta‐analysis. The pooled SMD of miR‐146a‐5p was −0.470 (95% CI: −0.902 to −0.038), *P* = 0.033, Fig. 5A) by the random‐effects model and the *P* value of the heterogeneity test was <0.001 (*I* ^2^ = 79%). The funnel plot shown in Fig. 5B did not imply significant publication bias (Begg's test: *P* = 0.917; Egger's test: *P* = 0.760).

**Table 1:** Summary of the included studies in the meta‐analysis

Study	HCC (*n*)	MiR‐146a‐5p expression		Nontumor (*n*)	MiR‐146a‐5p expression		*t*	*P*
		Mean	SD		Mean	SD		
GSE69580	5	9.8928	13.2138	5	3.4452	1.3785	1.085	0.309
GSE54751	10	0.1380	0.1312	10	0.1882	0.1004	−0.959	0.35
GSE41874	3	0.8520	0.1265	3	1.7150	0.2703	−5.008	0.007
GSE40744	39	9.9121	1.3010	18	9.8072	0.4518	0.448	0.656
GSE21362	73	6.4679	1.5814	73	7.5048	0.9066	−4.86	<0.001
GSE22058	96	0.9892	0.3288	96	1.26766	0.1114	−7.858	<0.001
GSE12717	10	10.7535	1.3380	6	10.7052	0.3907	0.085	0.933
GSE57555	5	−0.0419	0.0056	16	−0.0211	0.0253	−0.991	0.323
GSE10694	78	11.0580	0.5132	88	11.1343	0.4773	−1.796	0.088
Our combined data(2016)	89	0.7302	0.5142	89	1.3015	0.6934	−7.911	<0.001
TCGA(2016)	354	8.0304	1.6810	50	8.9665	0.8451	−6.274	<0.001

### TCGA RNA‐seq datasets

**Figure 6:**
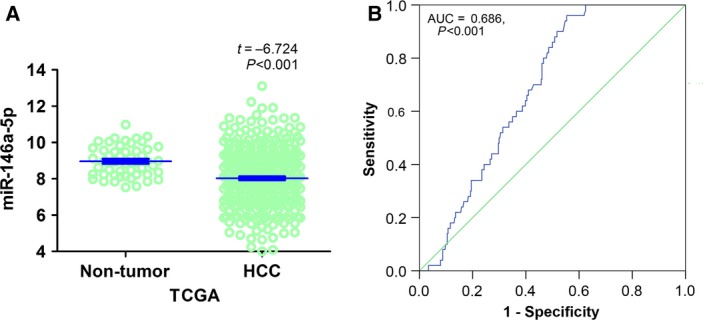
The miR‐146a‐5p expression data in HCC from TCGA datasets. (A) As for data gathered from the TCGA datasets, the miR‐146a‐5p expression in HCC was significantly decreased as compared to that in noncancerous liver tissues (8.0304 ± 1.6810 vs 8.9665 ± 0.8451, *t* = −6.274, *P* < 0.001). (B) The moderate diagnostic power of miR‐146a‐5p was identified from the receiver operator characteristic (ROC) curve based on TCGA data (AUC: 0.686, 95% CI: 0.628–0.744, *P* < 0.001).

For the RNA‐seq data, 377 randomized HCC tissues and 50 normal tissues were retrieved from the TCGA database (http://cancergenome.nih.gov/publications/publicationguidelines). The non‐HCC samples or samples with data deficiency were excluded; and 354 HCC patients were finally included in this study. Besides, the data of 50 normal liver tissues were retrieved for comparison. The expression of miR‐146a‐5p in HCC was down‐regulated (8.0304 ± 1.6810), as compared to its expression in normal liver tissues (8.9665 ± 0.8451, *t* = −6.274, *P* < 0.001, Fig. 6A). Moreover, a moderate diagnostic value of miR‐146a‐5p was identified via the receiver operator characteristic (ROC) curve and the area under the curve (AUC) was 0.686 (95% CI: 0.628–0.744, *P* < 0.001, Fig. 6B).

### Comprehensive meta‐analysis

**Figure 7:**
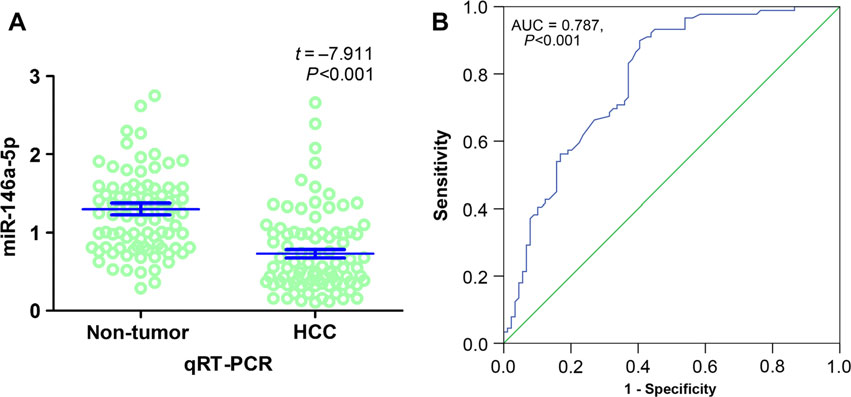
The miR‐146a‐5p expression data in HCC from qRT‐PCR. (A) The qRT‐PCR expression data, from our previous research with four newly added pairs, demonstrated that miR‐146a‐5p was significantly down‐regulated when compared to that in nontumor liver tissues (0.7302 ± 0.5142 vs 1.3015 ± 0.6934, *t* = −7.911, *P* < 0.001). (B) The AUC of miR‐146a‐5p here in ROC was 0.787 (95% CI: 0.720–0.854, *P* < 0.001).

After the search of electronic literature records, only one paper 9 formerly published by the current research group was found to be qualified according to the inclusion criteria. As previously reported 9, 85 HCC tissues with 85 corresponding adjacent nontumor liver tissues were investigated. In the current study, four new pairs of HCC and corresponding noncancerous tissues were included for the detection of miR‐146a‐5p expression with methods described previously 9. As can be expected, a lower level of miR‐146‐5p was observed in HCC tissues (0.7302 ± 0.5142) when compared with that in adjacent noncancerous liver tissues (1.3015 ± 0.6934, *t* = −7.911, *P* < 0.001, Fig. 7A). The AUC of miR‐146a‐5p in ROC was 0.787 (95% CI: 0.720–0.854, *P* < 0.001, Fig. 7B). The larger sample size of 89 tissue pairs was used for the following meta‐analysis.

**Figure 8:**
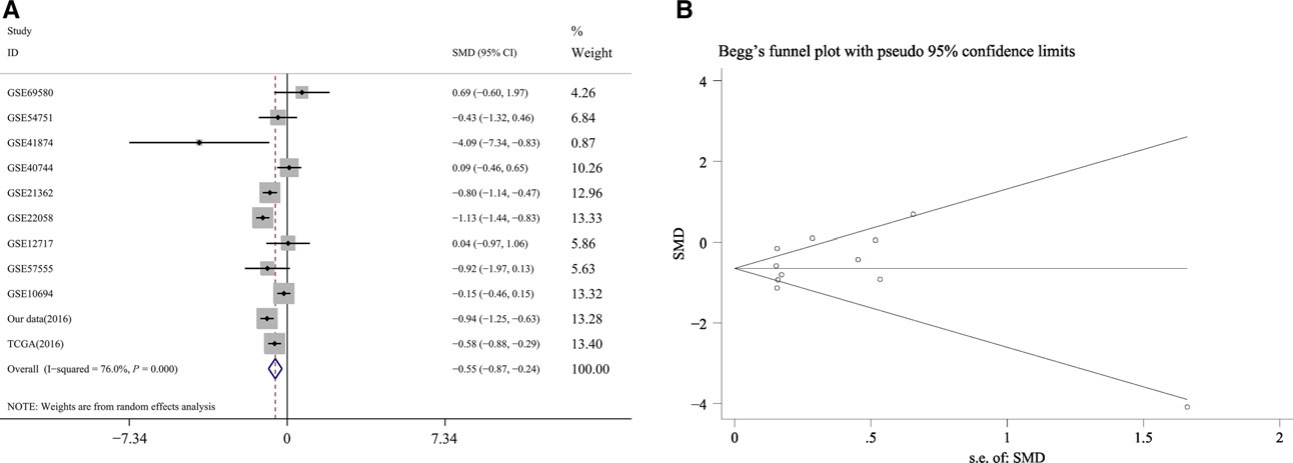
The forest plot and Begg's funnel plot of miR‐146a‐5p expression data from the most complete combination available of GEO database, TCGA dataset, our previous research article and four newly added pairs. (A) The miR‐146a‐5p expression data of 762 HCC and 454 noncancerous liver tissues from multiple resources were included. The pooled SMD of miR‐146a‐5p was −0.554 (95% CI: −0.866 to −0.241), *P* = 0.001) by the random‐effects model and the *P* value of the heterogeneity test was <0.001 (*I* ^2^ = 76%). (B) No publication bias was observed in the funnel plot (Begg's test: *P* = 0.876; Egger's test: *P* = 0.460).

The combination of 762 HCC and 454 noncancerous liver tissues was included for the meta‐analysis, which was from the various recourses such as GEO database, TCGA dataset, the previous article 9 and newly added samples and represents the most complete data available. The pooled SMD of miR‐146a‐5p was −0.554 (95% CI: −0.866 to −0.241), *P* = 0.001, Fig. 8A) by the random‐effects model and the *P* value of the heterogeneity test was <0.001 (*I* ^2^ = 76%). The funnel plot shown in Fig. 8B did not indicate publication bias (Begg's test: *P* = 0.876; Egger's test: *P* = 0.460). In summary, the current meta‐analysis further confirmed the down‐regulation of miR‐146a‐5p in HCC.

### Gene signatures of miR‐146a‐5p and HCC from the perspective of bioinformatics

#### NLP procedure of HCC

As formerly reported 15, a complete list of 64 577 entries of HCC‐related titles and abstracts was generated based on the literature from PubMed. The ensuing hypergeometric distribution algorithm featured 1800 HCC‐related genes 15.

#### Prediction of miR‐146a‐5p target genes

The prediction of miR‐146a‐5p target genes was performed with a union of 11 bioinformatics platforms as described. A certain gene would only be included if nominated by at least four prediction solutions. Accordingly, 251 genes were deemed eligible as potential miR‐146a‐5p target genes for the succeeding analyses.

#### Comprehensive integration

The comprehensive integration yielded a total of 104 genes (Table 2) by overlapping HCC‐related genes from NLP and miR‐146a‐5p potential target genes from prediction platforms.

**Table 2:** The comprehensive integration generated a total of 104 genes by overlapping HCC‐related genes from NLP and miR‐146a‐5p potential target genes from prediction platforms

Gene	*P* value	Gene description
ABCA1	0.022421	ATP‐binding cassette, subfamily A (ABC1), member 1
ABCC10	0.02059	ATP‐binding cassette, subfamily C (CFTR/MRP), member 10
ABCC11	0.03274	ATP‐binding cassette, subfamily C (CFTR/MRP), member 11
AFAP1L2	0.014458	Actin filament‐associated protein 1‐like 2
ANG	0.00060774	Angiogenin, ribonuclease, RNase A family, 5
APEX1	0.0010269	APEX nuclease (multifunctional DNA repair enzyme) 1
ARAF	0.074089	v‐raf murine sarcoma 3611 viral oncogene homolog
ATP7B	0.26196	ATPase, Cu++ transporting, beta polypeptide
BMP7	0.00080754	Bone morphogenetic protein 7
BNIP3	0.0047602	BCL2/adenovirus E1B 19 kDa interacting protein 3
BRCA1	0.65001	Breast cancer 1, early onset
BTG2	0.0035189	BTG family, member 2
C1ORF43	0.014458	Chromosome 1 open reading frame 43
CARD10	0.026684	Caspase recruitment domain family, member 10
CCL3	0.033443	Chemokine (C‐C motif) ligand 3
CCNA2	<1.00E‐08	Cyclin A2
CCNE2	0.00033179	Cyclin E2
CCT3	0.036756	Chaperonin containing TCP1, subunit 3 (gamma)
CD40LG	3.70E‐07	CD40 ligand
CDKN3	0.03475	Cyclin‐dependent kinase inhibitor 3
CFH	0.40925	Complement factor H
CHD1L	<1.00E‐08	Chromodomain helicase DNA‐binding protein 1‐like
CHEK1	0.00042545	CHK1 checkpoint homolog (S. pombe)
CHFR	0.076014	Checkpoint with forkhead and ring finger domains
CKAP4	0.00080042	Cytoskeleton‐associated protein 4
CNDP2	0.012405	CNDP dipeptidase 2 (metallopeptidase M20 family)
COMMD7	0.012405	COMM domain containing 7
COPS8	0.044739	COP9 constitutive photomorphogenic homolog subunit 8 (Arabidopsis)
CRY1	0.00097051	Cryptochrome 1 (photolyase‐like)
CTTN	<1.00E‐08	Cortactin
CYP2E1	0.005351	Cytochrome P450, family 2, subfamily E, polypeptide 1
DENND2D	0.010348	DENN/MADD domain containing 2D
EGFR	<1.00E‐08	Epidermal growth factor receptor (erythroblastic leukemia viral (v‐erb‐b) oncogene homolog, avian)
EIF5A2	3.16E‐06	Eukaryotic translation initiation factor 5A2
EPHA5	0.00097051	EPH receptor A5
ERBB4	0.19289	v‐erb‐a erythroblastic leukemia viral oncogene homolog 4 (avian)
FADD	<1.00E‐08	Fas (TNFRSF6)‐associated via death domain
FAS	2.16E‐08	Fas (TNF receptor superfamily, member 6)
FBXO8	0.01855	F‐box protein 8
FGB	0.35531	Fibrinogen beta chain
GALNT10	0.012405	UDP‐N‐acetyl‐alpha‐D‐galactosamine:polypeptide N‐acetylgalactosaminyltransferase 10 (GalNAc‐T10)
GNB2L1	0.0013975	Guanine nucleotide‐binding protein (G protein), beta polypeptide 2‐like 1
GPX3	0.0021848	Glutathione peroxidase 3 (plasma)
GTF2I	0.093154	General transcription factor II, i
HAS2	0.048705	Hyaluronan synthase 2
HNRNPD	0.11919	Heterogeneous nuclear ribonucleoprotein D (AU‐rich element RNA‐binding protein 1, 37 kDa)
IFI6	0.028707	Interferon, alpha‐inducible protein 6
IL3	0.13011	Interleukin 3 (colony‐stimulating factor, multiple)
IRAK1	0.17076	Interleukin‐1 receptor‐associated kinase 1
JMJD1A	0.016506	Jumonji domain containing 1A
KIF18A	0.030725	Kinesin family member 18A
KISS1	5.77E‐08	KiSS‐1 metastasis‐suppressor
KRT23	0.02059	Keratin 23 (histone deacetylase inducible)
LAMA2	0.076014	Laminin, alpha 2
LCK	0.47203	Lymphocyte‐specific protein tyrosine kinase
LIN28	<1.00E‐08	lin‐28 homolog (*Caenorhabditis elegans*)
LYZ	0.10626	Lysozyme (renal amyloidosis)
MARK2	0.0025961	MAP/microtubule affinity‐regulating kinase 2
MCPH1	0.054624	Microcephalin 1
MMP11	2.83E‐05	Matrix metallopeptidase 11 (stromelysin 3)
MST1R	0.093154	Macrophage‐stimulating 1 receptor (c‐met‐related tyrosine kinase)
MTA2	0.056589	Metastasis‐associated 1 family, member 2
MVD	0.01855	Mevalonate (diphospho) decarboxylase
NFE2	0.046724	Nuclear factor (erythroid‐derived 2), 45 kDa
NME1	<1.00E‐08	Nonmetastatic cells 1, protein (NM23A) expressed in
NODAL	0.042749	Nodal homolog (mouse)
NOX4	0.0053453	NADPH oxidase 4
NP	0.074089	Nucleoside phosphorylase
PA2G4	7.75E‐05	Proliferation‐associated 2G4, 38 kDa
PBLD	0.014458	Phenazine biosynthesis‐like protein domain containing
PDGFRB	1.82E‐06	Platelet‐derived growth factor receptor, beta polypeptide
PER3	3.49E‐05	Period homolog 3 (Drosophila)
PFTK1	0.024657	PFTAIRE protein kinase 1
PIWIL4	0.016506	piwi‐like 4 (Drosophila)
PLAUR	<1.00E‐08	Plasminogen activator, urokinase receptor
PLK2	0.036756	Polo‐like kinase 2 (Drosophila)
PMS1	0.0014645	PMS1 postmeiotic segregation increased 1 (*Saccharomyces cerevisiae*)
PPP2R4	<1.00E‐08	Protein phosphatase 2A activator, regulatory subunit 4
PRDX4	0.00038652	Peroxiredoxin 4
PSMD10	4.63E‐06	Proteasome (prosome, macropain) 26S subunit, non‐ATPase, 10
RAC2	0.0053453	ras‐related C3 botulinum toxin substrate 2 (rho family, small GTP‐binding protein Rac2)
ROCK1	3.31E‐07	Rho‐associated, coiled‐coil containing protein kinase 1
SLC1A5	0.052655	Solute carrier family 1 (neutral amino acid transporter), member 5
SMAD4	<1.00E‐08	SMAD family member 4
SNRPE	6.08E‐05	Small nuclear ribonucleoprotein polypeptide E
SORT1	0.077934	Sortilin 1
TFF3	0.0047602	Trefoil factor 3 (intestinal)
TGIF1	0.076014	TGFB‐induced factor homeobox 1
TLR3	4.45E‐07	Toll‐like receptor 3
TNFRSF13B	7.17E‐05	Tumor necrosis factor receptor superfamily, member 13B
TPT1	0.0035189	Tumor protein, translationally controlled 1
TRAF2	0.046769	TNF receptor‐associated factor 2
TRAF6	0.037075	TNF receptor‐associated factor 6
TRAV10	0.0020783	T‐cell receptor alpha variable 10
TSPAN1	<1.00E‐08	Tetraspanin 1
UHRF1	0.052655	Ubiquitin‐like with PHD and ring finger domains 1
VIM	<1.00E‐08	Vimentin
VWCE	6.44E‐05	von Willebrand factor C and EGF domains
WASF2	0.07985	WAS protein family, member 2
WNT3	0.00088342	Wingless‐type MMTV integration site family, member 3
XPC	0.0035853	Xeroderma pigmentosum, complementation group C
XRCC1	<1.00E‐08	X‐ray repair complementing defective repair in Chinese hamster cells 1
ZDHHC2	9.00E‐05	Zinc finger, DHHC‐type containing 2
ZNF23	0.01855	Zinc finger protein 23 (KOX 16)

#### Pathway enrichment analysis

Pathway enrichment analyses were performed for both miR‐146a‐5p predicted target genes and HCC‐related genes. For miR‐146a‐5p predicted target genes, a panel of 59 pathways (Table 3) was identified, among which four were deemed both statistically significant (*P* ≤ 0.05) and scientifically valuable, namely neurotrophin signaling pathway (count = 5; *P* = 0.000627; IRAK1, NRAS, RAC1, SORT1, TRAF6), adherens junction (count=4; *P* = 0.001949; RAC1, SMAD4, YES1, IQGAP1), VEGF signaling pathway (count=3; *P* = 0.025301; NRAS, PTGS2, RAC1), and toll‐like receptor (TLR) signaling pathway (count = 3; *P* = 0.043766; IRAK1, RAC1, TRAF6). As to HCC‐related genes obtained from NLP procedure, 24 statistically significant pathways (*P* ≤ 0.05) were reported previously 15.

**Table 3:** A panel of 59 pathways was identified for miR‐146a‐5p predicted target genes

Term	Count	*P* value	Genes
hsa04722: Neurotrophin signaling pathway	5	6.27E‐04	IRAK1, NRAS, RAC1, SORT1, TRAF6
hsa04520: Adherens junction	4	0.001949	RAC1, SMAD4, YES1, IQGAP1
hsa05200: Pathways in cancer	5	0.020692	NRAS, PTGS2, RAC1, SMAD4, TRAF6
hsa04370: VEGF signaling pathway	3	0.025301	NRAS, PTGS2, RAC1
hsa04620: Toll‐like receptor signaling pathway	3	0.043766	IRAK1, RAC1, TRAF6
hsa04360: Axon guidance	3	0.067759	NRAS, ROBO1, RAC1
hsa04810: Regulation of actin cytoskeleton	3	0.159939	NRAS, RAC1, IQGAP1
hsa05211: Renal cell carcinoma	2	0.210236	NRAS, RAC1
hsa05212: Pancreatic cancer	2	0.215582	RAC1, SMAD4
hsa04662: B cell receptor signaling pathway	2	0.223537	NRAS, RAC1
hsa05220: Chronic myeloid leukemia	2	0.223537	NRAS, SMAD4
hsa04010: MAPK signaling pathway	3	0.223547	NRAS, RAC1, TRAF6
hsa04664: Fc epsilon RI signaling pathway	2	0.231416	NRAS, RAC1
hsa05210: Colorectal cancer	2	0.246948	RAC1, SMAD4
hsa05222: Small cell lung cancer	2	0.246948	PTGS2, TRAF6
hsa04012: ErbB signaling pathway	2	0.254603	NRAS, ERBB4
hsa04650: Natural killer cell mediated cytotoxicity	2	0.363187	NRAS, RAC1
hsa04530: Tight junction	2	0.365373	NRAS, YES1
hsa04120: Ubiquitin‐mediated proteolysis	2	0.371889	PARK2, TRAF6
hsa04310: Wnt signaling pathway	2	0.401474	RAC1, SMAD4
hsa04144: Endocytosis	2	0.466104	ERBB4, TRAF6
hsa04062: Chemokine signaling pathway	2	0.471642	NRAS, RAC1
hsa04510: Focal adhesion	2	0.496777	TLN2, RAC1
hsa00270: Cysteine and methionine metabolism	1	>0.99	MTAP
hsa00590: Arachidonic acid metabolism	1	>0.99	PTGS2
hsa04020: Calcium signaling pathway	1	>0.99	ERBB4
hsa04060: Cytokine‐cytokine receptor interaction	1	>0.99	IL17A
hsa04110: Cell cycle	1	>0.99	SMAD4
hsa04142: Lysosome	1	>0.99	SORT1
hsa04210: Apoptosis	1	>0.99	IRAK1
hsa04320: Dorso‐ventral axis formation	1	>0.99	NOTCH2
hsa04330: Notch signaling pathway	1	>0.99	NOTCH2
hsa04350: TGF‐beta signaling pathway	1	>0.99	SMAD4
hsa04540: Gap junction	1	>0.99	NRAS
hsa04610: Complement and coagulation cascades	1	>0.99	CFH
hsa04621: NOD‐like receptor signaling pathway	1	>0.99	TRAF6
hsa04622: RIG‐I‐like receptor signaling pathway	1	>0.99	TRAF6
hsa04660: T‐cell receptor signaling pathway	1	>0.99	NRAS
hsa04666: Fc gamma R‐mediated phagocytosis	1	>0.99	RAC1
hsa04670: Leukocyte transendothelial migration	1	>0.99	RAC1
hsa04710: Circadian rhythm	1	>0.99	PER1
hsa04720: Long‐term potentiation	1	>0.99	NRAS
hsa04730: Long‐term depression	1	>0.99	NRAS
hsa04910: Insulin signaling pathway	1	>0.99	NRAS
hsa04912: GnRH signaling pathway	1	>0.99	NRAS
hsa04916: Melanogenesis	1	>0.99	NRAS
hsa05012: Parkinson's disease	1	>0.99	PARK2
hsa05014: Amyotrophic lateral sclerosis (ALS)	1	>0.99	RAC1
hsa05120: Epithelial cell signaling in Helicobacter pylori infection	1	>0.99	RAC1
hsa05213: Endometrial cancer	1	>0.99	NRAS
hsa05214: Glioma	1	>0.99	NRAS
hsa05215: Prostate cancer	1	>0.99	NRAS
hsa05216: Thyroid cancer	1	>0.99	NRAS
hsa05218: Melanoma	1	>0.99	NRAS
hsa05219: Bladder cancer	1	>0.99	NRAS
hsa05221: Acute myeloid leukemia	1	>0.99	NRAS
hsa05223: Nonsmall cell lung cancer	1	>0.99	NRAS
hsa05416: Viral myocarditis	1	>0.99	RAC1

#### Gene connectivity

**Figure 9:**
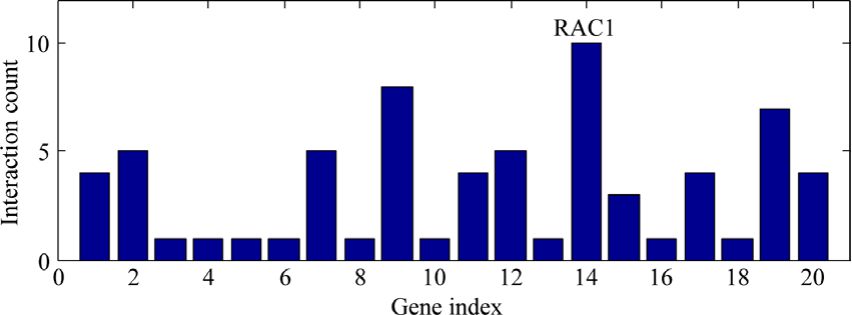
Gene connectivity test for miR‐146a‐5p predicted target genes. Gene connectivity test established the top gene connectivity of RAC1 (*z*‐test, *P* = 0.007305) among all the 20 hub genes of miR‐146a, interacting with 10 different genes in total.

The gene connectivity analysis provided us with a quantitative interface to understand the interacting degree of related genes and proteins. As to miR‐146a‐5p predicted target genes, the gene connectivity of RAC1 ranked top (*z*‐test, *P* = 0.007305, Fig. 9, Table 4) among all the 20 hub genes of miR‐146a‐5p, interacting with 10 different genes in total (ERBB4, IQGAP1, NRAS, PARK2, PTGS2, RACGAP1, ROBO1, SMAD4, TRAF6, YES1). For HCC‐related genes, the gene connectivity results were reported in our previous article 15.

**Table 4:** Results of gene connectivity test for miR‐146a‐5p predicted target genes

Gene	Degrees	*P* value	Interactions
RAC1	10	0.007305	ERBB4, IQGAP1, NRAS, PARK2, PTGS2, RACGAP1, ROBO1, SMAD4, TRAF6, YES1
NRAS	8	0.044385	ELAVL1, ERBB4, NOTCH2, PTGS2, RAC1, RACGAP1, SMAD4, YES1
TRAF6	7	0.091440	ERBB4, IL17A, IRAK1, OTUD7B, PARK2, RAC1, SORT1
ERBB4	5	0.276934	NOTCH2, NRAS, PTGS2, RAC1, TRAF6
NOTCH2	5	0.276934	ERBB4, HEYL, NRAS, PTGS2, SMAD4
PTGS2	5	0.276934	ELAVL1, ERBB4, NOTCH2, NRAS, RAC1
ELAVL1	4	0.412161	NOVA1, NRAS, PARK2, PTGS2
PARK2	4	0.412161	ELAVL1, RAC1, SMAD4, TRAF6
SMAD4	4	0.412161	NOTCH2, NRAS, PARK2, RAC1
YES1	4	0.412161	NRAS, PTPRE, RAC1, RACGAP1
RACGAP1	3	0.558826	NRAS, RAC1, YES1
HEYL	1	0.812719	NOTCH2
IL17A	1	0.812719	TRAF6
IQGAP1	1	0.812719	RAC1
IRAK1	1	0.812719	TRAF6
NOVA1	1	0.812719	ELAVL1
OTUD7B	1	0.812719	TRAF6
PTPRE	1	0.812719	YES1
ROBO1	1	0.812719	RAC1
SORT1	1	0.812719	TRAF6

#### Regulatory network

**Figure 10:**
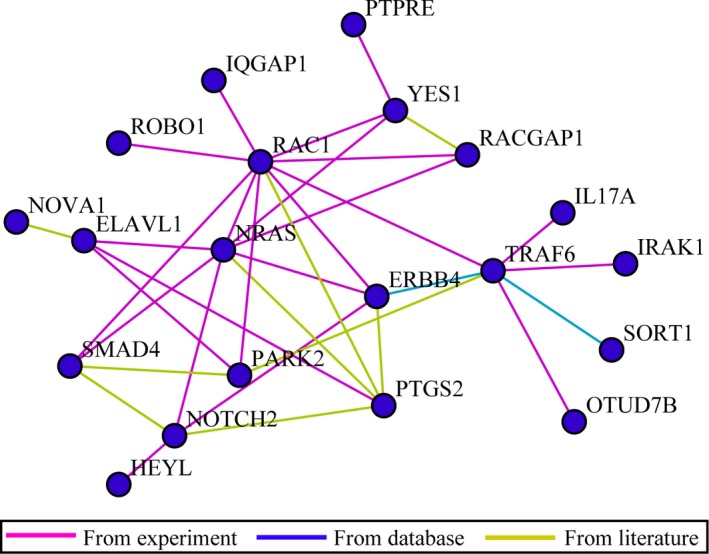
Regulatory network construction for miR‐146a‐5p predicted target genes. Regulatory network was constructed to unveil the potential regulatory network of miR‐146a‐5p.

**Figure 11:**
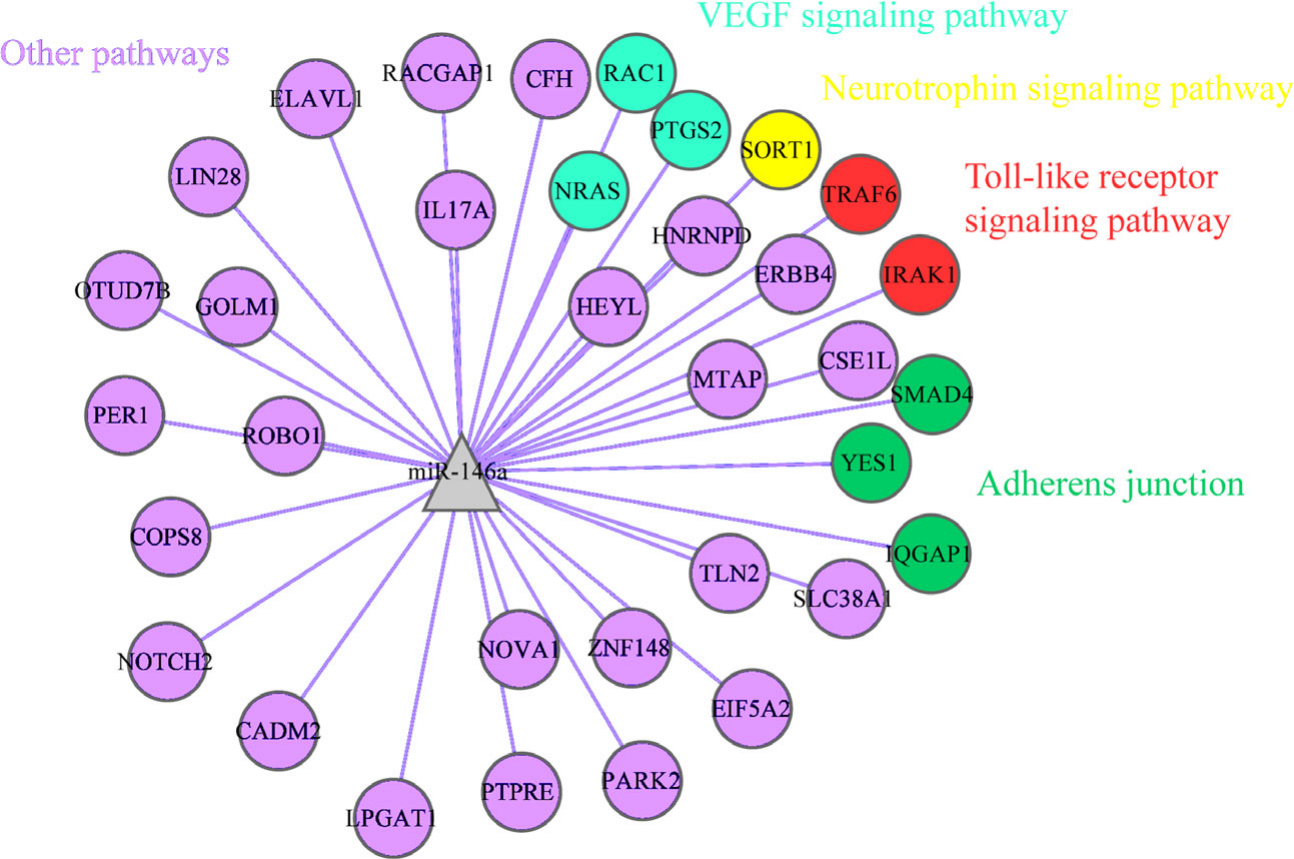
Regulatory network construction for the overlapped genes. miR‐146a‐5p might interact with RAC1, PTGS2, and NRAS via VEGF signaling pathway and mediate biological processes with SMAD4, YES1 and IQGAP1 via adherens junction. SORT1 might be associated with miR‐146a‐5p via neurotrophin signaling pathway and Toll‐like receptor signaling pathway could be in charge for the interactions and regulations between miR‐146a and TRAF6 and IRAK1. The rest of genes might interact with miR‐146a‐5p via various pathways.

Regulatory networks were constructed for miR‐146a‐5p predicted target genes (Fig. 10), HCC‐related genes 15, and the overlapping genes (Fig. 11), respectively. With regard to the overlapping genes, which are considered to be both miR‐146a‐5p and HCC‐related, miR‐132 might interact with RAC1, PTGS2, and NRAS via VEGF signaling pathway and mediate biological processes with SMAD4, YES1, and IQGAP1 via adherens junction. SORT1 might be associated with miR‐146a‐5p via neurotrophin signaling pathway and TLR signaling pathway could be in charge for the interactions and regulations between miR‐146a‐5p and TRAF6 and IRAK1. The rest of genes might interact with miR‐146a‐5p via various pathways.

## Discussion

Hepatocellular carcinoma takes the third place for cancer‐related deaths and is the fifth most frequent type of cancer internationally 1, 2. To make things worse, delayed diagnosis, recurrence, and metastasis shatter the treatment opportunities for HCC patients 3. miRs are considered to be actively involved in numerous oncological processes 4, 5, and the conspicuous miR‐146a‐5p 6, 7, 8 is one of them.

It has been reported in our previous article 9 that miR‐146a‐5p is decreased and might play a tumor‐suppressive role in HCC. However, we believed that it would be of great merit to validate the down‐expression of miR‐146a‐5p in HCC with a larger cohort and that the potential molecular mechanisms and regulatory networks should be unveiled, which led to the current study.

In the present study, we took great advantage of the GEO and TCGA databases to gather massive data of miR‐146a‐5p expression in HCC and later performed a meta‐analysis with the GEO‐based microarrays, TCGA‐based RNA‐seq data, previous research 9, and four newly added tissue pairs in order to validate the miR‐146a‐5p down‐expression and to explore its diagnostic values in HCC. Furthermore, miR‐146a‐5p potential target genes were predicted on 11 bioinformatics solutions. With the formerly reported HCC‐related genes from NLP, we were able to develop the HCC‐ and miR‐146a‐5p‐related overlaps analytically. Last but not least, comprehensive analyses were conducted for the above genes in an attempt to discover the gene signatures and potential regulatory pathways and networks of miR‐146a‐5p in HCC.

In relation to the data acquisition, we employed the collaboration of GEO, TCGA, and reported qRT‐PCR as well as newly included pairs. In GEO Database, we included nine microarrays with 319 HCC and 315 noncancerous liver tissues (GSE69580, GSE54751, GSE41874, GSE40744, GSE21362, GSE22058, GSE12717, GSE57555, and GSE10694) after rigorous screening procedures. Three microarrays (GSE41874, GSE21362, and GSE22058) presented statistically significant results that the miR‐146a‐5p expression was decreased in HCC tissues as compared to the noncancerous counterparts. Two microarrays (GSE21362 and GSE22058) demonstrated the significant diagnostic value of miR‐146a‐5p in HCC (AUC = 0.749, 95% CI: 0.669–0.830, *P* < 0.001; AUC = 0.801, 95% CI: 0.731–0.872, *P* < 0.001, respectively). Meanwhile, in TCGA Database, the RNA‐seq data of 354 randomized HCC and 50 normal tissues were finally included. The miR‐146a‐5p expression in HCC was significantly down‐regulated as compared to its expression in normal liver tissues (8.0304 ± 1.6810 vs 8.9665 ± 0.8451, *t* = −6.274, *P* < 0.001). Furthermore, a moderate diagnostic value of miR‐146a‐5p in HCC was perceived via ROC (AUC: 0.686; 95% CI: 0.628–0.744, *P* < 0.001). Besides, a new combined cohort using qRT‐PCR was established by pooling data from the previous research 9 and four newly included pairs. Unsurprisingly, the decreased expression of miR‐146‐5p was observed in HCC tissues (0.7302 ± 0.5142) when compared with that in adjacent noncancerous liver tissues (1.3015 ± 0.6934, *t* = −7.911, *P* < 0.001) and the remarkable diagnostic value of miR‐146a‐5p in HCC should be denoted (AUC: 0.787; 95% CI: 0.720–0.854, *P* < 0.001).

For the comprehensive meta‐analysis, a large cohort of 762 HCC and 454 noncancerous liver tissues from GEO database, TCGA dataset, the previous article, 9 and four newly added samples were included, which renders the integrated perspective with the most complete data. A pooled SMD of −0.554 (95% CI: −0.866 to −0.241), *P* = 0.001) was presented by the random‐effects model. The *P* value of the heterogeneity test was <0.001 (*I* ^2^ = 76%) and no publication bias was observed (Begg's test: *P* = 0.876; Egger's test: *P* = 0.460). Based on the above in the meta‐analysis, we can safely reach the conclusion that miR‐146a‐5p is down‐regulated in HCC, which is consistent with our previous findings 9.

The integrated computation consists of three parts; the natural language processing, the miR‐146a‐5p target genes’ prediction, and the comprehensive integration of overlapped genes. The NLP 15 established a record of 64 577 entries on the basis of the literature from PubMed, 1800 of which were proved HCC related by hypergeometric distribution. As to miR‐146a‐5p target genes’ prediction, an unprecedented union of 11 prediction tools was employed, and 251 genes were considered to be potential miR‐146a‐5p target genes since they were nominated by at least four platforms. Eventually, the comprehensive integration yielded a total of 104 genes by overlapping results from NLP and gene prediction, which were deemed both HCC‐ and miR‐146a‐5p related.

Three comprehensive bioinformatics analyses were applied in the study; pathway enrichment test, gene connectivity test, and regulatory network construction. First of all, four pathways were highlighted among all the 59 pathways identified in the pathway enrichment analysis for miR‐146a‐5p predicted target genes; that is, neurotrophin signaling pathway (count = 5; *P* = 0.000627; IRAK1, NRAS, RAC1, SORT1, TRAF6), adherens junction (count = 4; *P* = 0.001949; RAC1, SMAD4, YES1, IQGAP1), VEGF signaling pathway (count = 3; *P* = 0.025301; NRAS, PTGS2, RAC1), and TLR signaling pathway (count = 3; *P* = 0.043766; IRAK1, RAC1, TRAF6). In the connectivity test for miR‐146a‐5p, RAC1 ranked top as the most interacted gene among all the 20 hub genes of miR‐146a‐5p (z‐test, *P* = 0.007305), interplaying with 10 other genes altogether (*ERBB4, IQGAP1, NRAS, PARK2, PTGS2, RACGAP1,ROBO1, SMAD4, TRAF6*, and *YES1*). Regulatory networks constructed for the overlapping genes demonstrated that miR‐132 in HCC might interact with RAC1, PTGS2, and NRAS via VEGF signaling pathway, mediate biological processes with SMAD4, YES1, and IQGAP1 via adherens junction, associate with TRAF6 and IRAK1 via TLR signaling pathway, and interplay with SORT1 via neurotrophin signaling pathway.

The gene with top connectivity for miR‐146a‐5p, RAC1, and four pathways with high enrichment for the overlapped genes are worthy of extra attention since they might provide unique insights into molecule‐based diagnostic and therapeutic strategies.

The RAC1 gene encodes the Rac1 protein, which has been reported to be significantly regulatory in cell growth and motility specifically 30 and might result in metastasis, invasion 31, and epithelial mesenchymal transition (EMT) 32 in the oncological context. In HCC, the up‐regulation of Tiam1 and Rac1 has been found to associate with poor prognosis 33. Our previous research showed that miR‐146a‐5p expression was related to clinical TNM stage and metastasis 9. The relations between other miRs and RAC1 gene in HCC have already been extensively studied. Zhou *et al*. 34 reported that miR‐100 suppresses HCC metastasis by abolishing the ICMT‐Rac1 signaling and that the decrease in miR‐100 might promote the metastasis in HCC. Also, miR‐142‐3p is found to play a directly negative regulatory role of human RAC1 and able to suppress the HCC cell migration and invasion 35. The two studies 34, 35 mutually complement and support our current and previous 9 research in a sense: for one thing, the top connectivity highlighted the possible role of RAC1 in the down‐regulation of miR‐146a‐5p in HCC; for another, the two studies 34, 35 established the validated role of RAC1 in miR‐related HCC and proved the vigorous validity of bioinformatics tools in the present research, rendering us the potential role of RAC1 in miR‐146a‐5p‐related HCC. Considering the above, we speculate that RAC1 might be negatively regulated by miR‐146a‐5p and promote metastasis in miR‐146a‐5p‐related HCC.

The VEGF signaling pathway is considered to play a significant role in vasculogenesis and angiogenesis 36 by boosting the vascular permeability, proliferation and migration 37. The negative correlations 9 between miR‐146a‐5p and portal vein tumor embolus as well as metastasis might be related to VEGF signaling pathway. Recent research 7 proved that miR‐146a‐5p limits metastasis by down‐regulating VEGF in HCC, which supports our bioinformatics findings. Adherens junction is crucially associated with intercellular adhesion and is responsible for maintaining cell polarity and structures, which represses cell migration and proliferation 38. Thus, adherens junction might be positively regulated by miR‐146a‐5p and correlate with favorable clinical outcomes. TLR are widely expressed by various cells and play a significant part in inflammation and immune responses. Changes in TLR activities might exert the antitumor influence on HCC cells, which might prove useful for novel targeted therapeutics in HCC 39. Research has shown that the TLR pathway is involved in the initiation, progression, and metastasis of HCC and that the essential role of TLR4 should not be ignored in the pathogenesis and progression of HCC 40. Furthermore, studies 41, 42, 43 have shown the involvement of TLR and TLR pathway in miR‐146a‐5p‐related HCC or other liver diseases, which illuminate the potential regulatory mechanisms of miR‐146a‐5p and TLR in HCC. Neurotrophin signaling pathway is thought to be actively involved in many biological processes in the nervous system, with the examples of neurocyte development and higher order behaviors such as learning and memory. Neurotrophin signaling pathway and related microRNAs mutually regulate each other and are considered highly related to multiple cancers and brain diseases, whose mechanisms are auspicious in developing novel diagnostic and therapeutic strategies 44. The network construction for the overlaps implied that SORT1 and miR‐146a‐5p might interact via the neurotrophin signaling pathway.

There are three articles by Zhou *et al*. 34, Wu *et al*. 35, and Zhang *et al*. 7, respectively, that are particularly noteworthy, since they prove the validity and feasibility of the current bioinformatics analyses to a certain extent. Zhou *et al*. 34 stated that miR‐100 suppresses HCC metastasis by abolishing the ICMT‐Rac1 signaling and negatively correlates with metastasis in HCC, whereas Wu *et al*. argued that miR‐142‐3p negatively regulates RAC1 and is able to suppress the migration and invasion of HCC cells 35, both of which happened to cover the involvements of certain individual microRNAs in HCC and merit attention for the further verification of RAC1 and miR‐146a‐5p in HCC. Even more delightfully, Zhang *et al*. 7 reported that miR‐146a‐5p confines metastasis by negatively regulating VEGF in HCC, which has precisely verified the bioinformatics discovery—the same microRNA (miR‐146a‐5p) exerts its influence in the same disease (HCC) via the exactly same identified pathway (VEGF signaling pathway).

Several aspects of the study add to its merits. First of all, the study gathered the most complete data currently available (762 HCC vs 454 noncancerous tissues) and validated the down‐regulation of miR‐146a‐5p in HCC, which is the first of its kind. Secondly, the study provided us with the gene signatures for HCC‐ and miR‐146a‐5p‐related overlapped genes, mapping out the potentially primary picture for the further exploration into the mechanisms. Thirdly, the powerful combination of 11 bioinformatics tools for prediction maximized the reliability of miR‐146a‐5p target gene prediction results, since other previous articles only used the utmost of three prediction platforms. Last but not least, the predicted target genes of miR‐146a‐5p and the related informatics analyses have all been made available in the current article along with the supplementary files, which are easily accessible and reusable for further study purposes. Still, *in vitro* experimental validation and verification are needed for the featured miR‐146a‐5p hub gene with top connectivity, RAC1, as well as the four identified pathways with their interacting genes, which the team plans to perform in future.

## Conclusions

The study gathered the most complete data currently available from multiple resources (GEO‐based microarrays, TCGA‐based RNA‐seq data, and qRT‐PCR), validated the down‐regulation of miR‐146a‐5p and denoted its diagnostic values in HCC. An outstanding union of 11 bioinformatics platforms predicted a total of 251 potential target genes of miR‐146a‐5p. With the HCC‐related genes from NLP, the overlaps of 104 genes were generated, which are considered both HCC‐ and miR‐146a‐5p related. Last but not least, the bioinformatics analyses highlighted RAC1 as the most connected hub gene for miR‐146a‐5p and four pathways with high enrichment were featured for the overlapped genes, both of which could prove useful for future molecule‐based diagnostics and therapeutics of HCC.

## Author contributions

XZ, YD, and GC conceived and designed the experiments. XZ, ZY, HL, FR, PL, YD, and GC performed the experiments. XZ, ZY, HL, FR, PL, YD, and GC analyzed the data. XZ, ZY, HL, FR, PL, YD, and GC contributed reagents/materials/analysis tools. XZ, YD, and GC wrote the manuscript. All authors read and approved the final manuscript.

## Supporting information


**Table S1.** List of all the 251 potential miR‐146a‐5p target genes. A total of 251 genes were predicted by at least four bioinformatics platforms, making them eligible potential miR‐146a‐5p target genes.
