# Supplementary material for: Down‐regulation of miR‐146a‐5p and its potential targets in hepatocellular carcinoma validated by a TCGA‐ and GEO‐based study
Source: FEBS Open Bio. 2017 Feb 20;7(4):504–21. doi: 10.1002/2211-5463.12198 (PMC5377416; doi:10.1002/2211-5463.12198)
Supplement: Supplementary file 1 — Table S1. List of all the 251 potential miR‐146a‐5p target genes. A total of 251 genes were predicted by at least four bioinformatics platforms, making them eligible potential miR‐146a‐5p target genes. [file FEB4-7-504-s001.pdf]

Target Genes	Total Counts	Description
USP3	6	ubiquitin specific peptidase 3
SYT1	6	synaptotagmin I
CCDC117	6	coiled-coil domain containing 117
FBXL10	6	F-box and leucine-rich repeat protein 10
BCORL1	6	BCL6 co-repressor-like 1
HMBOX1	6	homeobox containing 1
SFRS6	6	splicing factor, arginine/serine-rich 6
SMAD4	6	SMAD family member 4
PRX	5	periaxin
SYNPR	5	synaptoporin
SORT1	5	sortilin 1
KCTD15	5	potassium channel tetramerisation domain containing 15
ZNF512B	5	zinc finger protein 512B
PSMD3	5	proteasome (prosome, macropain) 26S subunit, non-ATPase, 3
BIVM	5	basic, immunoglobulin-like variable motif containing
LRRC15	5	leucine rich repeat containing 15
ZAK	5	sterile alpha motif and leucine zipper containing kinase AZK
STC1	5	stanniocalcin 1
SEC23IP	5	SEC23 interacting protein
C5orf23	5	chromosome 5 open reading frame 23
IRAK1	5	interleukin-1 receptor-associated kinase 1
NOVA1	5	neuro-oncological ventral antigen 1
PAPPA	5	pregnancy-associated plasma protein A, pappalysin 1
PCDH1	5	protocadherin 1
PIP4K2B	5	phosphatidylinositol-5-phosphate 4-kinase, type II, beta
PHOX2B	5	paired-like homeobox 2b
SP8	5	Sp8 transcription factor
ZDHHC17	5	zinc finger, DHHC-type containing 17

Target Genes	Total Counts	Description
FLJ20309	5	hypothetical protein FLJ20309
RAB10	5	RAB10, member RAS oncogene family
OTUD4	5	OTU domain containing 4
THRB	5	thyroid hormone receptor, beta (erythroblastic leukemia viral (v-erb-a) oncogene homolog 2, avian)
DTNA	5	dystrobrevin, alpha
C6orf203	5	chromosome 6 open reading frame 203
CASK	5	calcium/calmodulin-dependent serine protein kinase (MAGUK family)
STX3	5	syntaxin 3
NPAS4	5	neuronal PAS domain protein 4
EGR3	5	early growth response 3
STRBP	5	spermatid perinuclear RNA binding protein
C6orf97	4	chromosome 6 open reading frame 97
NOTCH2	4	Notch homolog 2 (Drosophila)
KIAA1715	4	KIAA1715
PARD6G	4	par-6 partitioning defective 6 homolog gamma (C. elegans)
WWC2	4	WW and C2 domain containing 2
SLC19A3	4	solute carrier family 19, member 3
HNRNPD	4	heterogeneous nuclear ribonucleoprotein D (AU-rich element RNA binding protein 1, 37kDa)
PID1	4	phosphotyrosine interaction domain containing 1
SNX22	4	sorting nexin 22
ZC3H8	4	zinc finger CCCH-type containing 8
LIN28	4	lin-28 homolog (C. elegans)
BHLHB3	4	basic helix-loop-helix domain containing, class B, 3
ITCH	4	itchy E3 ubiquitin protein ligase homolog (mouse)
KIAA1826	4	KIAA1826
MPPE1	4	metallophosphoesterase 1

Target Genes	Total Counts	Description
SEMA3G	4	sema domain, immunoglobulin domain (Ig), short basic domain, secreted, (semaphorin) 3G
KLHL4	4	kelch-like 4 (Drosophila)
TXNDC10	4	thioredoxin domain containing 10
KIAA1199	4	KIAA1199
MARK1	4	MAP/microtubule affinity-regulating kinase 1
C3orf10	4	chromosome 3 open reading frame 10
LIN7C	4	lin-7 homolog C (C. elegans)
C9orf72	4	chromosome 9 open reading frame 72
TMEM19	4	transmembrane protein 19
ZNF532	4	zinc finger protein 532
PAPD1	4	PAP associated domain containing 1
USP47	4	ubiquitin specific peptidase 47
ST7L	4	suppression of tumorigenicity 7 like
GIPC2	4	GIPC PDZ domain containing family, member 2
GOLM1	4	golgi membrane protein 1
OTUD7B	4	OTU domain containing 7B
MRS2	4	MRS2 magnesium homeostasis factor homolog (S. cerevisiae)
ZNF649	4	zinc finger protein 649
BCL11A	4	B-cell CLL/lymphoma 11A (zinc finger protein)
RFXDC2	4	regulatory factor X domain containing 2
SLC25A14	4	solute carrier family 25 (mitochondrial carrier, brain), member 14
ZNF148	4	zinc finger protein 148
SRR	4	serine racemase
ZFYVE1	4	zinc finger, FYVE domain containing 1
PMAIP1	4	phorbol-12-myristate-13-acetate-induced protein 1
ZNF253	4	zinc finger protein 253
ZBTB2	4	zinc finger and BTB domain containing 2

Target Genes	Total Counts	Description
CRAMP1L	4	Crm, cramped-like (Drosophila)
PTGFRN	4	prostaglandin F2 receptor negative regulator
EIF5A2	4	eukaryotic translation initiation factor 5A2
PLSCR4	4	phospholipid scramblase 4
RAB8B	4	RAB8B, member RAS oncogene family
14-Sep	4	septin 14
PPP2R5C	4	protein phosphatase 2, regulatory subunit B″, gamma isoform
SOX5	4	SRY (sex determining region Y)-box 5
FMNL3	4	formin-like 3
RUNX1T1	4	runt-related transcription factor 1; translocated to, 1 (cyclin D-related)
JAZF1	4	JAZF zinc finger 1
DPY19L2	4	dpy-19-like 2 (C. elegans)
MOBKL1A	4	MOB1, Mps One Binder kinase activator-like 1A (yeast)
ZFP91-CNTF	4	ZFP91-CNTF
IGSF1	4	immunoglobulin superfamily, member 1
NRP2	4	neuropilin 2
PMEPA1	4	prostate transmembrane protein, androgen induced 1
RAC1	4	ras-related C3 botulinum toxin substrate 1 (rho family, small GTP binding protein Rac1)
PAQR9	4	progestin and adipoQ receptor family member IX
HIPK1	4	homeodomain interacting protein kinase 1
COPS8	4	COP9 constitutive photomorphogenic homolog subunit 8 (Arabidopsis)
ZNF605	4	zinc finger protein 605
C12orf36	4	chromosome 12 open reading frame 36
THAP5	4	THAP domain containing 5
NF2	4	neurofibromin 2 (merlin)
SYT14	4	synaptotagmin XIV
RPESP	4	RPE-spondin

Target Genes	Total Counts	Description
THAP3	4	THAP domain containing, apoptosis associated protein 3
PTPRE	4	protein tyrosine phosphatase, receptor type, E
COX15	4	COX15 homolog, cytochrome c oxidase assembly protein (yeast)
ZNF354B	4	zinc finger protein 354B
TIFA	4	TRAF-interacting protein with forkhead-associated domain
RPL34	4	ribosomal protein L34
FBXO4	4	F-box protein 4
TRIM5	4	tripartite motif-containing 5
VASN	4	vasorin
CADM2	4	cell adhesion molecule 2
MS4A1	4	membrane-spanning 4-domains, subfamily A, member 1
ZNF449	4	zinc finger protein 449
SLC16A14	4	solute carrier family 16, member 14 (monocarboxylic acid transporter 14)
PQLC3	4	PQ loop repeat containing 3
TRAF6	4	TNF receptor-associated factor 6
FUK	4	fucokinase
RBM11	4	RNA binding motif protein 11
ZNF558	4	zinc finger protein 558
SAMD8	4	sterile alpha motif domain containing 8
TBC1D20	4	TBC1 domain family, member 20
AMPH	4	amphiphysin
SLC1A1	4	solute carrier family 1 (neuronal/epithelial high affinity glutamate transporter, system Xag), member 1
PER1	4	period homolog 1 (Drosophila)
NRAS	4	neuroblastoma RAS viral (v-ras) oncogene homolog
MTAP	4	methylthioadenosine phosphorylase
IL17A	4	interleukin 17A
CNTFR	4	ciliary neurotrophic factor receptor

Target Genes	Total Counts	Description
ELAVL1	4	ELAV (embryonic lethal, abnormal vision, Drosophila)-like 1 (Hu antigen R)
CSE1L	4	CSE1 chromosome segregation 1-like (yeast)
CLCN6	4	chloride channel 6
CASP7	4	caspase 7, apoptosis-related cysteine peptidase
BCAT2	4	branched chain aminotransferase 2, mitochondrial
TBX18	4	T-box 18
ZNF331	4	zinc finger protein 331
SLC38A1	4	solute carrier family 38, member 1
PPBP	4	pro-platelet basic protein (chemokine (C-X-C motif) ligand 7)
PTPRA	4	protein tyrosine phosphatase, receptor type, A
EFNB2	4	ephrin-B2
PRPF4B	4	PRP4 pre-mRNA processing factor 4 homolog B (yeast)
IQGAP1	4	IQ motif containing GTPase activating protein 1
WDR22	4	WD repeat domain 22
KLF7	4	Kruppel-like factor 7 (ubiquitous)
ARSB	4	arylsulfatase B
PIP5K1B	4	phosphatidylinositol-4-phosphate 5-kinase, type I, beta
C2orf3	4	chromosome 2 open reading frame 3
SVIL	4	supervillin
SH3GL2	4	SH3-domain GRB2-like 2
ROBO1	4	roundabout, axon guidance receptor, homolog 1 (Drosophila)
RNF4	4	ring finger protein 4
STIL	4	SCL/TAL1 interrupting locus
ERBB4	4	v-erb-a erythroblastic leukemia viral oncogene homolog 4 (avian)
RECQL5	4	RecQ protein-like 5
FLJ45831	4	FLJ45831 protein
SPOPL	4	speckle-type POZ protein-like
ZDHHC13	4	zinc finger, DHHC-type containing 13

Target Genes	Total Counts	Description
PTGS2	4	prostaglandin-endoperoxide synthase 2 (prostaglandin G/H synthase and cyclooxygenase)
GRIA3	4	glutamate receptor, ionotrophic, AMPA 3
GAD1	4	glutamate decarboxylase 1 (brain, 67kDa)
GABRA1	4	gamma-aminobutyric acid (GABA) A receptor, alpha 1
CCK	4	cholecystokinin
CD79B	4	CD79b molecule, immunoglobulin-associated beta
PLP1	4	proteolipid protein 1 (Pelizaeus-Merzbacher disease, spastic paraplegia 2, uncomplicated)
PHKB	4	phosphorylase kinase, beta
CFH	4	complement factor H
DLGAP1	4	discs, large (Drosophila) homolog-associated protein 1
ZNF813	4	zinc finger protein 813
EIF4G2	4	eukaryotic translation initiation factor 4 gamma, 2
NSL1	4	NSL1, MIND kinetochore complex component, homolog (S. cerevisiae)
OPALIN	4	oligodendrocytic myelin paranodal and inner loop protein
SDHC	4	succinate dehydrogenase complex, subunit C, integral membrane protein, 15kDa
CUGBP2	4	CUG triplet repeat, RNA binding protein 2
ACTBL2	4	actin, beta-like 2
SYT14L	4	synaptotagmin XIV-like
SPIN4	4	spindlin family, member 4
ZC3H12B	4	zinc finger CCCH-type containing 12B
C1orf69	4	chromosome 1 open reading frame 69
SPOP	4	speckle-type POZ protein
NUMB	4	numb homolog (Drosophila)
OR9Q1	4	olfactory receptor, family 9, subfamily Q, member 1
TOR1A	4	torsin family 1, member A (torsin A)
VPS54	4	vacuolar protein sorting 54 homolog (S. cerevisiae)

Target Genes	Total Counts	Description
SLITRK3	4	SLIT and NTRK-like family, member 3
RHOBTB3	4	Rho-related BTB domain containing 3
LPGAT1	4	lysophosphatidylglycerol acyltransferase 1
RNF10	4	ring finger protein 10
KIAA0141	4	KIAA0141
BZW1	4	basic leucine zipper and W2 domains 1
UBXD8	4	UBX domain containing 8
HEYL	4	hairy/enhancer-of-split related with YRPW motif-like
ARFGAP3	4	ADP-ribosylation factor GTPase activating protein 3
ZNF229	4	zinc finger protein 229
TNRC6A	4	trinucleotide repeat containing 6A
SLC39A1	4	solute carrier family 39 (zinc transporter), member 1
ADAMTS3	4	ADAM metallopeptidase with thrombospondin type 1 motif, 3
C16orf72	4	chromosome 16 open reading frame 72
ZNF365	4	zinc finger protein 365
PDS5B	4	PDS5, regulator of cohesion maintenance, homolog B (S. cerevisiae)
RARB	4	retinoic acid receptor, beta
PHF20L1	4	PHD finger protein 20-like 1
UBR5	4	ubiquitin protein ligase E3 component n-recognin 5
LRRTM2	4	leucine rich repeat transmembrane neuronal 2
FNBP4	4	formin binding protein 4
TRIM37	4	tripartite motif-containing 37
WDR7	4	WD repeat domain 7
TMEM194A	4	transmembrane protein 194A
POFUT2	4	protein O-fucosyltransferase 2
ANKRD12	4	ankyrin repeat domain 12
ANKRD28	4	ankyrin repeat domain 28
CLASP2	4	cytoplasmic linker associated protein 2

Target Genes	Total Counts	Description
HIC2	4	hypermethylated in cancer 2
TLN2	4	talin 2
RACGAP1	4	Rac GTPase activating protein 1
NPTN	4	neuroplastin
GJC1	4	gap junction protein, gamma 1, 45kDa
RND2	4	Rho family GTPase 2
YES1	4	v-yes-1 Yamaguchi sarcoma viral oncogene homolog 1
ST5	4	suppression of tumorigenicity 5
PRKCE	4	protein kinase C, epsilon
MAP3K8	4	mitogen-activated protein kinase kinase kinase 8
MED13	4	mediator complex subunit 13
MYO6	4	myosin VI
ONECUT2	4	one cut homeobox 2
DLGAP2	4	discs, large (Drosophila) homolog-associated protein 2
PARK2	4	Parkinson disease (autosomal recessive, juvenile) 2, parkin
NEB	4	nebulin
MYT1	4	myelin transcription factor 1
LRP2	4	low density lipoprotein-related protein 2
PRMT3	4	protein arginine methyltransferase 3
CD96	4	CD96 molecule
FBXO3	4	F-box protein 3
FBXW2	4	F-box and WD repeat domain containing 2
SRP72	4	signal recognition particle 72kDa
SFRS1	4	splicing factor, arginine/serine-rich 1 (splicing factor 2, alternate splicing factor)
NFAT5	4	nuclear factor of activated T-cells 5, tonicity-responsive
ZNF138	4	zinc finger protein 138
MXD4	4	MAX dimerization protein 4

Target Genes	Total Counts	Description
DLC1	4	deleted in liver cancer 1
SIRPB1	4	signal-regulatory protein beta 1
LANCL1	4	LanC lantibiotic synthetase component C-like 1 (bacterial)
MMP16	4	matrix metallopeptidase 16 (membrane-inserted)
MAT2A	4	methionine adenosyltransferase II, alpha
6-Mar	4	membrane-associated ring finger (C3HC4) 6
TRDMT1	4	tRNA aspartic acid methyltransferase 1
